# GPGPS: a robust prognostic gene pair signature of glioma ensembling *IDH* mutation and 1p/19q co-deletion

**DOI:** 10.1093/bioinformatics/btac850

**Published:** 2023-01-13

**Authors:** Lixin Cheng, Haonan Wu, Xubin Zheng, Ning Zhang, Pengfei Zhao, Ran Wang, Qiong Wu, Tao Liu, Xiaojun Yang, Qingshan Geng

**Affiliations:** Shenzhen People’s Hospital, First Affiliated Hospital of Southern University of Science and Technology, Second Clinical Medicine College of Jinan University, Shenzhen 518020, China; Shenzhen People’s Hospital, First Affiliated Hospital of Southern University of Science and Technology, Second Clinical Medicine College of Jinan University, Shenzhen 518020, China; Department of Geriatrics, Shenzhen Clinical Research Center for Aging, Shenzhen 518020, China; Shenzhen People’s Hospital, First Affiliated Hospital of Southern University of Science and Technology, Second Clinical Medicine College of Jinan University, Shenzhen 518020, China; Department of Computer Science and Engineering, The Chinese University of Hong Kong, Shatin, New Territories, Hong Kong; Guangdong Provincial Key Laboratory of Infectious Disease and Molecular Immunopathology, Shantou University Medical College, Shantou 515041, China; Shenzhen People’s Hospital, First Affiliated Hospital of Southern University of Science and Technology, Second Clinical Medicine College of Jinan University, Shenzhen 518020, China; Department of Geriatrics, Shenzhen Clinical Research Center for Aging, Shenzhen 518020, China; Shenzhen People’s Hospital, First Affiliated Hospital of Southern University of Science and Technology, Second Clinical Medicine College of Jinan University, Shenzhen 518020, China; Department of Computer Science and Engineering, The Chinese University of Hong Kong, Shatin, New Territories, Hong Kong; Hong Kong Genome Institute, Shatin, New Territories, Hong Kong; International Digital Economy Academy, Shenzhen 518020, China; Guangdong Provincial Key Laboratory of Infectious Disease and Molecular Immunopathology, Shantou University Medical College, Shantou 515041, China; Shenzhen People’s Hospital, First Affiliated Hospital of Southern University of Science and Technology, Second Clinical Medicine College of Jinan University, Shenzhen 518020, China; Department of Geriatrics, Shenzhen Clinical Research Center for Aging, Shenzhen 518020, China

## Abstract

**Motivation:**

Many studies have shown that *IDH* mutation and 1p/19q co-deletion can serve as prognostic signatures of glioma. Although these genetic variations affect the expression of one or more genes, the prognostic value of gene expression related to *IDH* and 1p/19q status is still unclear.

**Results:**

We constructed an ensemble gene pair signature for the risk evaluation and survival prediction of glioma based on the prior knowledge of the *IDH* and 1p/19q status. First, we separately built two gene pair signatures *IDH*-GPS and 1p/19q-GPS and elucidated that they were useful transcriptome markers projecting from corresponding genome variations. Then, the gene pairs in these two models were assembled to develop an integrated model named Glioma Prognostic Gene Pair Signature (GPGPS), which demonstrated high area under the curves (AUCs) to predict 1-, 3- and 5-year overall survival (0.92, 0.88 and 0.80) of glioma. GPGPS was superior to the single GPSs and other existing prognostic signatures (avg AUC = 0.70, concordance index = 0.74). In conclusion, the ensemble prognostic signature with 10 gene pairs could serve as an independent predictor for risk stratification and survival prediction in glioma. This study shed light on transferring knowledge from genetic alterations to expression changes to facilitate prognostic studies.

**Availability and implementation:**

Codes are available at https://github.com/Kimxbzheng/GPGPS.git

**Supplementary information:**

Supplementary data are available at *Bioinformatics* online.

## 1 Introduction

Gliomas are the most common primary malignant central nervous system (CNS) tumor, the incidence of which ranges from five to six cases per 100 000 population in most countries. Every year, approximately 100 000 diffuse glioma patients increase worldwide (Bray *et al.*, 2018). In the 2016 edition of World Health Organization (WHO) classification of CNS tumors, the classification of diffused gliomas is based on presence/absence of isocitrate dehydrogenase (*IDH*) mutation and complete deletion of both the short arm of chromosome 1 (1p) and the long arm of chromosome 19 (19q) [1p/19q co-deletion (1p/19q-cd)] (Eckel-Passow *et al.*, 2015; Kawaguchi *et al.*, 2016). Histologically, the *IDH* wild-type (*IDH*-wt) and the *IDH*-mutant, 1p/19q-non-co-deleted diffuse gliomas generally are astrocytoma, while the *IDH*-mutant, 1p/19q-co-deleted tumors are oligodendroglioma (Wesseling and Capper, 2018).

Numerous targeted and large-scale studies have demonstrated that *IDH* mutation and 1p/19q-cd are the well-recognized prognostic factors in glioma that often lead to high sensitivity to chemotherapy (Cairncross *et al.*, 2013). Many models of gene signatures based on *IDH* mutation and 1p/19q-cd status can predict prognosis and be employed as an independent prognostic factor (Hu *et al.*, 2017; Wu *et al.*, 2019; Zhang *et al.*, 2019; Zuo *et al.*, 2019). However, predicting prognosis using the 1p/19q-cd or *IDH* mutation is time-consuming and it usually takes a week to obtain the result. Gene expression is an important molecular phenotype that associates genetic variation with phenotypic variation, and changes in gene regulation play a central role in determining differences in gene expression (Andersen *et al.*, 2008). So we attempted to quantify the survival risk at the transcriptome level by transferring the information from genome alterations to gene pair expression changes, which would substantially reduce the detection time and benefit the establishment of therapeutic strategy.

Currently, numerous studies on the prediction modeling of diseases are based on the gene expression value (Cheng *et al.*, 2020; Liu *et al.*, 2020; Yang *et al.*, 2021). Frequently, using the absolute abundance of gene expression may lead to biased results because biological variations are inevitable between different physiological statuses (Cheng *et al.*, 2016a, b; Liu *et al.*, 2019). Moreover, different detection platforms and pre-processing methods have a huge impact on the subsequent analysis results (Li *et al.*, 2022). The mainstream normalization methods usually assume that the gene expressions are similarly or even identically distributed in each sample, regardless of the variance between different statuses, for example, mutant or wild-type, resulting in inaccurate quantification of genes and affecting the subsequent differential analysis (Li *et al.*, 2022; Liu *et al.*, 2019). To this end, we previously developed a method, individualized Pairwise Analysis of Gene Expression (iPAGE), to identify gene pairs using the relative expression value based on internal comparisons with the sample, which is not affected by the normalization methods (Wang *et al.*, 2022; Wu *et al.*, 2022; Zheng *et al.*, 2021). iPAGE sophisticatedly compared the rank of a pair of genes in an individual sample and selected those gene pairs significantly invert from one class to the other as relevant features. It is based on a mild and universally satisfied assumption that the normalization procedure is monotonic in the expression values, resulting iPAGE adapts to most types of omics data as long as the quantification value is monotonic.

We believe that an appropriate method is capable of well representing the projection from genome to transcriptome, such as from gene mutation to gene expression. In this study, we assume that the expression changes of gene pairs at the transcriptome level can respond to IDH mutation or 1p/19q-cd at the genome level. The integrated gene pairs representing both IDH mutation and 1p/19q-cd are expected to achieve a more robust performance, which has significant instruction for prognosis evaluation and subsequent treatment. We separately select the reverse gene pairs with significant expression change for IDH mutations and 1p/19q-cd and construct gene pair signatures for them based on two cohorts TCGA and GSE16011. Then, the prognostic value of the two signatures is evaluated in four independent cohorts from the CGGA and Gene Expression Omnibus (GEO) databases. After that, the prognostic value of our signature and the potential biological functions associated with this signature have been systematically studied.

## 2 Materials and methods

### 2.1 Transcriptome data

The mRNA expression cohorts of glioma patients were acquired from The Cancer Genome Atlas program (TCGA, https://tcga-data.nci.nih.gov/tcga) and Chinese Glioma Genome Atlas (CGGA, http://www.cgga.org.cn/). In addition, using the keywords ‘glioma’, ‘*IDH1*’ and ‘1p/19q’ for human mRNA dataset searching, we obtained three cohorts GSE16011, GSE43113 and GSE43388 from database GEO (Barrett *et al.*, 2013) and E-TABM-3892 from ArrayExpress (Sarkans *et al.*, 2021). Both low grade glioma (LGG) and glioblastoma multiforme (GBM) were included (Zhao *et al.*, 2021). The clinical information including *IDH1* mutation and 1p/19q-cd were also collected. A detailed description for each cohort was listed in Table 1. No normalization methods were carried out and only the raw data were used for gene pair selection. The main outcome of our study was overall survival (OS).

**Table 1.** Transcriptome datasets with genetic variation information

Cohorts	Platforms	Sample size	LGG	GBM	IDH mut	1p/19q codel	Both
Discovery							
TCGA	Illumina HiSeq 2000	451	441	–	361	146	146
GSE16011	HG-U133_Plus_2	285	117	159	83	45	25
Validation							
E-TABM-3892	HG-U133_Plus_2	179	148	21	133	99	96
CGGA	Illumina HiSeq 2000/2500	325	181	144	158	67	57
GSE43113	HG-U133_Plus_2	28	25	1	11	10	7
GSE43388	HG-U133_Plus_2	43	41	2	44	36	21

### 2.2 Gene pair detection and model construction

**Fig. 1.**
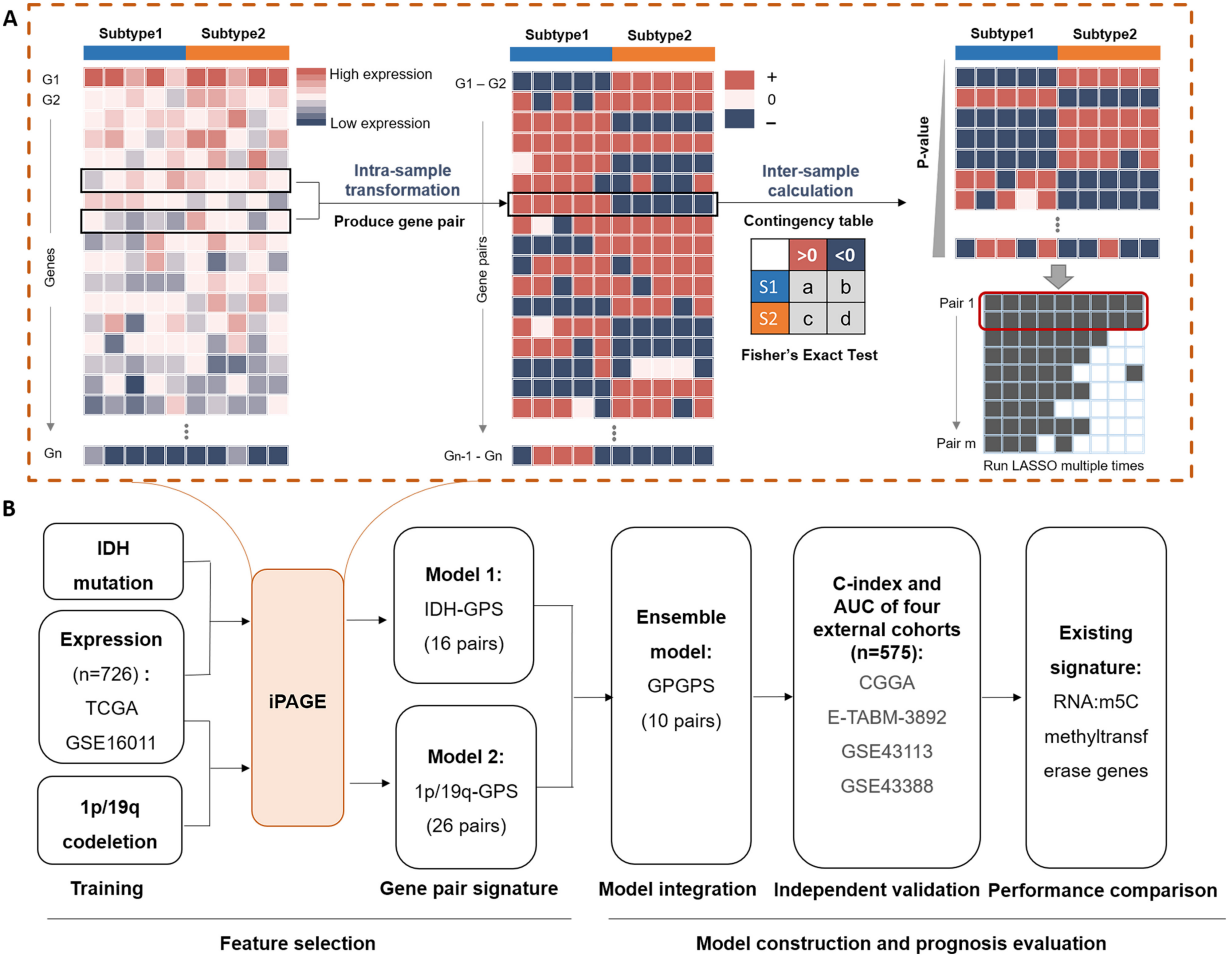
Workflow of this study. (**A**) The procedure of iPAGE. Individual gene profile was first transferred to gene pair profile (intra-sample transformation), and then the gene pairs with significant changes between subtypes were identified using Fisher’s exact test (inter-sample calculation). LASSO was finally used to build the gene pair signatures. (**B**) Flowchart presenting the process of establishing the gene pairs to construct and evaluate the prognostic model. IDH-GPS and 1p/19q-GPS were combined to ensemble a more accurate model GPGPS using a forward selection procedure

We considered the effects of genetic variation on gene expression in the brain tissue of glioma patients. To obtain the relative expression order of gene pairs, we transferred the absolute gene expression value to the order of gene for each sample. Then, we calculated the mean rank deviations between the *IDH*-mt and *IDH*-wt samples (Fig. 1A). The top 10% up-regulated genes (gene rank in *IDH*-mt higher than *IDH*-wt) and the top 10% down-regulated genes (gene rank in *IDH*-mt lower than *IDH*-wt) with the largest rank difference were used to construct gene pairs. Next, we evaluated the reverse degree of gene pairs using Fisher’s exact test and 50 out of them with the lowest *P*-value were defined as reverse gene pairs. For a given gene pair, *G_i_* and *G_j_*, either in the *IDH*-mt group or in the *IDH*-wt group, we computed the number of four types of gene pairs, *G_i_ > G_j_* in *IDH*-wt, *G_i_ < G_j_* in *IDH*-wt, *G_i_ > G_j_* in *IDH*-mt and *G_i_ < G_j_* in *IDH*-mt. Fisher’s exact test was then used to estimate the statistical significance.

After that, a least absolute shrinkage and selection operator (LASSO) logistic regression algorithm was used to optimally identify a panel of reverse gene pairs that are significantly associated with *IDH* mutation (Fig. 1B). Then, we performed LASSO 50 times and the reverse gene pairs consistently identified were used to construct the final prognostic model. After determining the gene pair panel, logistic regression analysis was used to assign the contribution weight and generate the risk score model, which is able to stratify the patients into low- and high-risk groups based on an optimum cutoff. Simultaneously, the samples with 1p/19q-cd information were processed in the same way. The identified gene pair signatures were named *IDH*-GPS and 1p/19q-GPS, respectively.

### 2.3 Model ensembling

The reverse gene pairs of *IDH*-GPS and 1p/19q-GPS were combined into a new set and we refined them to further identify gene pairs with the optimal classification effect of high-risk and low-risk groups using a forward selection procedure. The forward selection algorithm starts with the most significant feature in the model and then keeps adding features one by one until the model achieves the best performance. The ranked features are added in sequence and the model is refitted with these features (Qi *et al.*, 2016). The advantage of forward selection is that it usually generates small models with less susceptible to collinearity, resulting in a prognostic model with only a few features and simple decision rules. Ultimately, we built an ensemble prognostic risk score model using the optimized gene pairs and if more than half of the gene pairs in the panel are reversed, a patient is predicted as high risk. Otherwise, it is a low-risk patient with a favorable prognosis.

### 2.4 Characterization of reverse gene pairs

The expression levels of the reverse gene pairs in the ensemble model were visualized in a heatmap. ‘RCircos’ package provided the information of chromosome location. Gene Set Enrichment Analysis (GSEA) was performed to reveal enriched pathways related to the risk stratification, a *P*-value < 0.05 was considered as statistically significant. R package ‘maftools’ was used to compare the mutation frequency of genes related to glioma between the high- and low-risk groups.

### 2.5 Survival analysis and prognosis validation

R packages ‘survminer’ and ‘survival’ were used to create survival curves and the concordance index (C-index). According to the risk score, patients were divided into high- and low-risk groups separately based on the optimal value. For the ensemble model, we set 0.5 as the cutoff value to categorize the patients into low-risk and high-risk groups. Kaplan–Meier curves were drawn to compare the OS differences between the high-risk and low-risk groups using the log-rank test (Cheng and Leung, 2018). Receiver operating characteristic (ROC) curves and the area under the curve (AUC) values were used to evaluate the ability of the gene pair signatures to represent *IDH* mutation status and 1p/19q-cd status. We also used the ROC curves to compare the prognostic accuracy in predicting 1-, 3- and 5-year OS between different models. The hazard ratio (HR) was calculated by Cox proportional hazards regression analysis to describe the relative risk of prognostic models. All the above calculations were conducted using R 4.0.3.

### 2.6 External validation of the prognostic signature

Wang *et al.* (2020) developed a glioma prognostic signature consisting of 5-methylcytosine (m5C) methyltransferases-related genes by using absolute gene expression values. To compare with this model, we calculated the AUC of 1-, 3- and 5-year OS predictions and used Kaplan–Meier to measure the fraction of high-risk group and low-risk group. Similarly, the ROC curves of 1-, 3- and 5-year OS and Kaplan–Meier curves of m5C methyltransferases model and our composite model were compared to determine the performance of the risk signature.

## 3 Results

### 3.1 Projection from genome to transcriptome

**Fig. 2.**
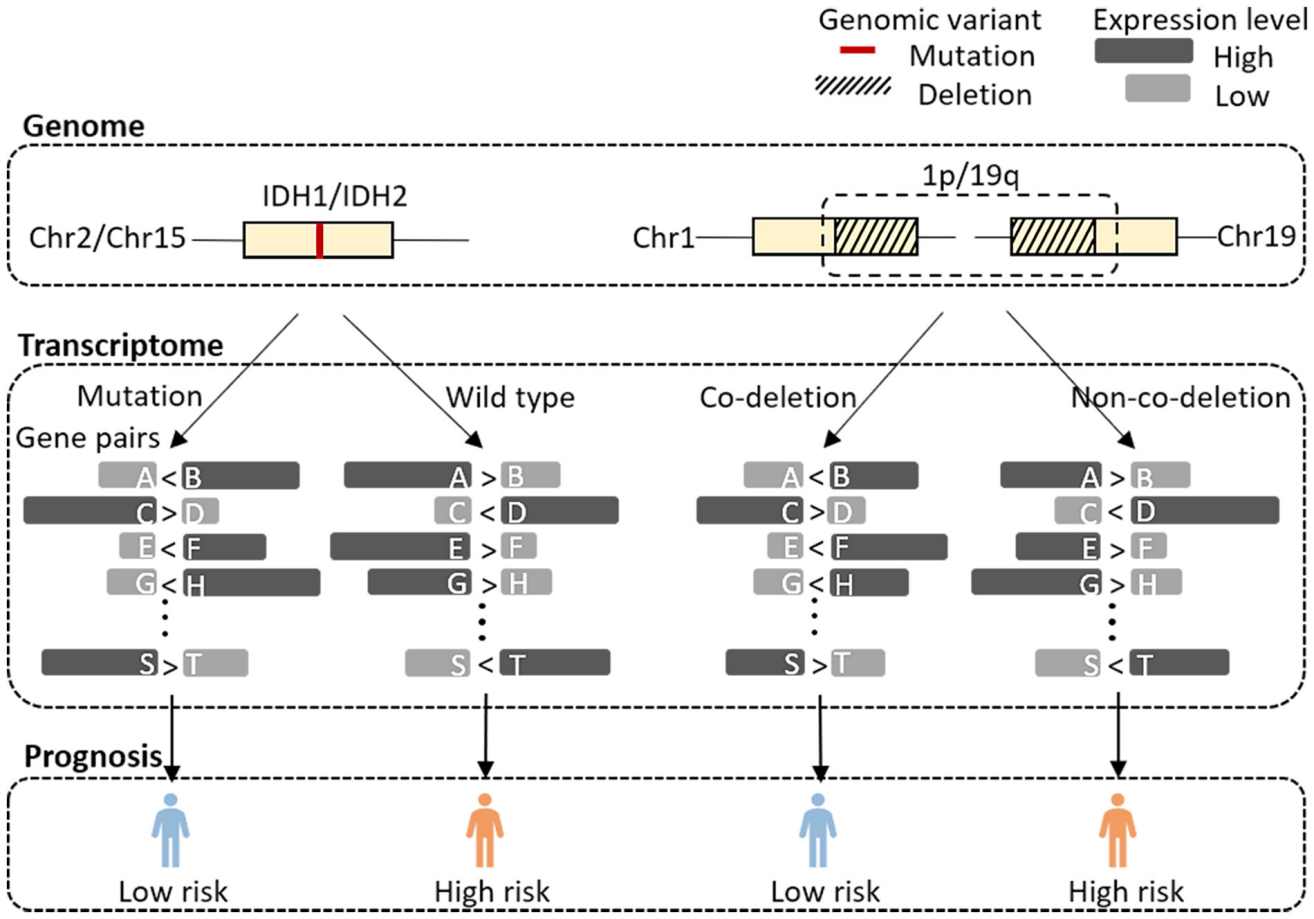
Diagram of the projection from genome to transcriptome for *IDH* mutation and 1p/19q-cd

We aimed to identify a panel of gene expression pairs that are significantly associated with a genetic variation, which is a projection from genome to transcriptome. Specifically, to represent the status of *IDH* mutation at the transcriptome level, we identified gene pairs whose expression level was significantly reverse between the mutant *IDH* group and wild-type *IDH* group (Fig. 2, left panel). For a pair of genes, for instance, gene *i* and gene *j*, the expression of gene *i* is larger than gene *j* (*G_i_ > G_j_*) in a majority of the wild-type *IDH* samples, while the order is reverse in a majority of the mutant *IDH* samples (*G_i_* < *G_j_*). Not limited to gene mutation, other genomic variants such as chromosome co-deletion can also be represented at the transcriptome level using gene pairs (Fig. 2, right panel). Mathematically, we build a classifier consisting of only a few gene pairs to discriminate samples with different genomic status.

*IDH* mutation and 1p/19q-cd are the two most important genetic factors for glioma prognosis, so we separately project them to the gene expression pairs and built gene pair signatures and then ensemble the signatures to build a powerful prognostic model.

### 3.2 Construction of gene pair signature

To build an effective prognostic model, we systematically collected the expression profiles and clinical information of glioma patients from TCGA and CCGA. Four cohorts from the GEO database were also used, including GSE16011, GSE61374, GSE43113 and GSE43388. The TCGA and GSE16011 cohorts with the largest size of LGG and GBM were combined and used as the training set, which consists of 736 patients with clinical information, including *IDH* mutant (*IDH*-mt, *n* = 442) and *IDH*-wt (*n* = 215) as well as 1p/19q-cd (*n* = 409) and non-co-deletion (1p/19q-ncd, *n* = 191). The other four cohorts were used as external validation sets (Fig. 1B). iPAGE is originally designed to screen gene pairs of interest for predictive model construction. The method only considers the relative expression value based on internal comparisons within the sample, not affected by the normalization methods and batch effect across datasets and platforms, which can serve as an alternative strategy for data integration.

Using iPAGE, we identified 2187 gene pairs that were significantly reversed between the *IDH*-mt and *IDH*-wt groups based on the training set. For instance, RBP1 and ATOH8 (*G_i_* and *G_j_*) are the most significantly reversed gene pair (*P*-value < 3.50*e*−95, Fisher’s exact test). The numbers of *G_i_ > G_j_* in *IDH*-wt, *G_i_ < G_j_* in *IDH*-wt, *G_i_ > G_j_* in *IDH*-mt and *G_i_ < G_j_* in *IDH*-mt were 193, 22, 39 and 403, respectively (see Methods). Among these gene pairs, 16 were selected by LASSO regression algorithm. Moreover, we identified 1879 gene pairs that were significantly reversed between the 1p/19q-cd and 1p/19q-ncd group, and 26 pairs out of them were further selected using LASSO. These two gene panels were defined as *IDH* mutation gene pair signature (*IDH*-GPS) and 1p/19q-cd gene pair signature (1p/19q-GPS), respectively.

### 3.3 Evaluation of gene pair signature

**Fig. 3.**
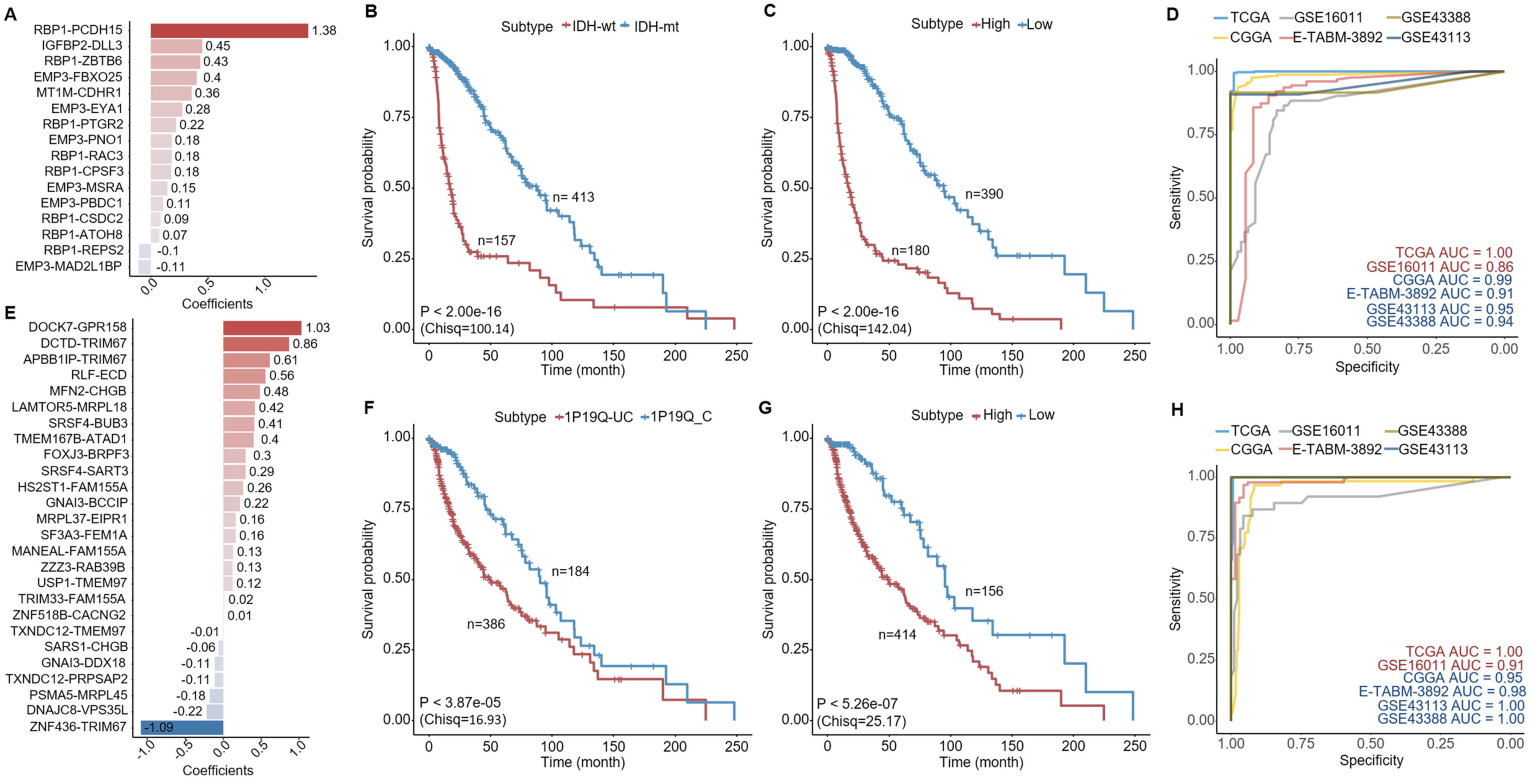
Performance evaluation of the *IDH* and 1p/19q GPS. (**A**) Bar plot demonstrating the coefficients of 16 gene pairs in *IDH* status. (**B**) Kaplan–Meier curves showing the OS of two patient groups with and without IDH mutation. (**C**) Survival analysis for the two patient groups in high and low risk predicted by the *IDH*-GPS. (**D**) ROC curves showing the performance of the *IDH*-GPS in discriminating different subtypes, *IDH*-mt and wild-type. (**E**) Bar plot demonstrating the coefficients of 26 gene pair in 1p/19q status. (**F**) Survival analysis for the two patient groups with and without 1p/19q-cd. (**G**) Survival analysis for the two patient groups in high and low risk predicted by the 1p/19q-GPS. (**H**) The performance of the 1p/19q-GPS in different datasets

Among the 16 gene pairs of *IDH*-GPS, *RBP1* was included in eight of them and EMP3 was involved in six pairs (Fig. 3A), suggesting that *RBP1* and *EMP3* play a vital role in responding to *IDH* mutation. As expected, the two groups of glioma patients, *IDH*-mt and *IDH*-wt, demonstrated a significant difference in survival analysis (Fig. 3B). Specifically, the *IDH*-mt group showed better OS than the *IDH*-wt (log-rank *P*-value < 2.0*e*−16). Next, to better understand the relationship between the gene pairs and the survival time, we calculated individualized risk scores of the two groups of patients using *IDH*-GPS and reassigned them into high- and low-risk groups based on the average risk score. A significant difference in OS was also presented between the two groups (log-rank *P*-value < 2.0*e*−16; Fig. 3C). Subsequently, five external cohorts were used to evaluate whether GPS was a generalized model that could represent the status of *IDH* mutation. The *IDH*-GPS model demonstrated that AUCs ranged from 0.86 to 1 across the five cohorts, indicating the gene pairs have a promising ability to discriminate the *IDH*-mt from the *IDH*-wt patients (Fig. 3D).

Simultaneously, 26 gene pairs were selected to construct the 1p/19q-GPS signature and no genes appeared multiple times (Fig. 3E). We carried out survival analysis for the 1p/19q-cd and 1p/19q-ncd patients and evaluated their prognostic risk using 1p/19q-GPS. The same conclusion was drawn and distinct separation in survival outcomes was yielded (Fig. 3F and G). Surprisingly, the 1p/19q-GPS model yielded AUCs of over 0.91 around all cohorts (Fig. 3H). Overall, these findings elucidated a decent projection from *IDH* mutation (or 1p/19q-cd) at the genome level to expression changes at the transcriptome level leveraging reverse gene pairs.

### 3.4 Construction and characterization of the ensemble model

**Fig. 4.**
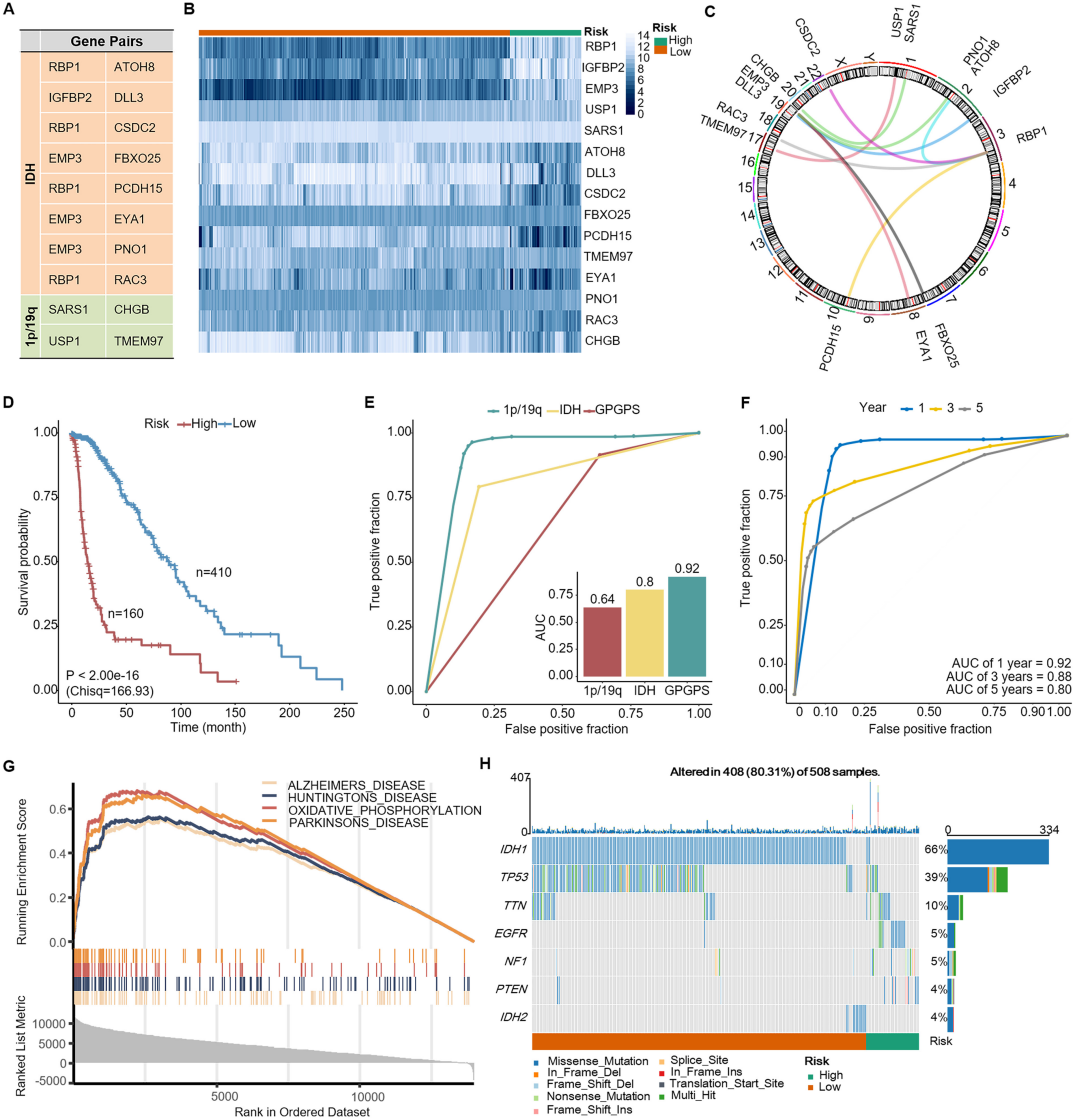
Construction and characterization of GPGPS. (**A**) Gene pair composition of GPGPS. (**B**) Expressions of the genes of GPGPS in the high- and low-risk groups. (**C**) Circos plot presents the position in chromosome of each gene pair. (**D**) Kaplan–Meier analysis of OS for low-risk and high-risk patients in the training cohort. High risk or low risk was defined based on the GPGPS model. (**E**) The ROC curves for 1-year OS predictions of the three risk models, *IDH*-GPS, 1p/19q-GPS and GPGPS. (**F**) The ROC curves of the ensemble model for 1-, 3- and 5-year OS predictions. (**G**) GSEA enrichment analysis showing the 10 gene pairs were significantly involved in the neurodegenerative disorder pathways. (**H**) Waterfall plot representing the gene mutation frequency between the high- and low-risk groups

Ultimately, the 16 *IDH* mutation gene pairs and the 26 1p/19q-cd gene pairs were assembled to develop an integrated model, glioma prognostic Gene Pair Signature (GPGPS). The architecture of GPGPS involves two base models, *IDH*-GPS and 1p/19q-GPS, and it stacks the predictions of both of them using the majority vote classifier. Ten gene pairs were identified, including eight *IDH*-GPS gene pairs and two 1P/19Q-GPS gene pairs, that is, *RBP1-ATOH8*, *IGFBP2-DLL3*, *RBP1-CSDC2*, *EMP3-FBXO25*, *RBP1-PCDH15*, *EMP3-EYA1*, *EMP3-PNO1*, *RBP1-RAC3*, *SARS1-CHGB* and *USP1-TMEM97* (Fig. 4A and Supplementary Fig. S1). Most of the genes showed distinct expression patterns between the high- and low-risk groups (Fig. 4B). The chromosome positions of these gene pairs were illustrated in the circos plot (Fig. 4C). According to the majority vote rule, patients with more than half of gene pairs (five pairs) voted were determined as high risk, and vice versa.

Survival analysis was carried out for the GPGPS model and the resulting Kaplan–Meier curves depicted that patients with higher risk scores had worse clinical outcomes and vice versa (Fig. 4D). The GPGPS model demonstrated an AUC of 0.92, which is much higher than the other two basic models (0.8 and 0.64) to predict 1-year OS (Fig. 4E). For the 3- and 5-year OS, the ensemble risk model also yielded acceptable AUCs (0.88 and 0.807) (Fig. 4F).

### 3.5 Risk stratification of GPGPS in TCGA

Next, we attempted to demonstrate whether the TCGA patients were well stratified by the risk scores of GPGPS in terms of functions. We investigated the implications of biological pathways for the expression difference between the high- and low-risk groups of patients using the TCGA gene expression data. The GSEA result illustrated that the enriched pathways are mainly associated with neurodegenerative disorders, including Alzheimer’s disease, Huntington’s disease and Parkinson’s disease (Fig. 4G). Fold change between the high- and low-risk groups was used to rank the gene list. In these pathways, the dysregulated genes tend to up-regulate in the high-risk groups.

We also carried out the mutation landscape analysis of glioma based on the risk stratification of GPGPS (Fig. 4H). The most frequently mutated genes in glioma are *IDH1*, *TP53*, *TTN*, *EGFR*, *NF1*, *PTEN* and *IDH2*. According to the risk stratification of GPGPS, *IDH1* and *IDH2* were mutant in a majority of the low-risk patients, indicating that these two genes can lead to a lower risk of glioma and GPGPS is qualified to represent the mutant status of *IDH*. For the 100 samples not illustrated in the mutation profile, most of them present 1p/19q-cd (data not shown).

### 3.6 Evaluation of the ensemble model

**Fig. 5.**
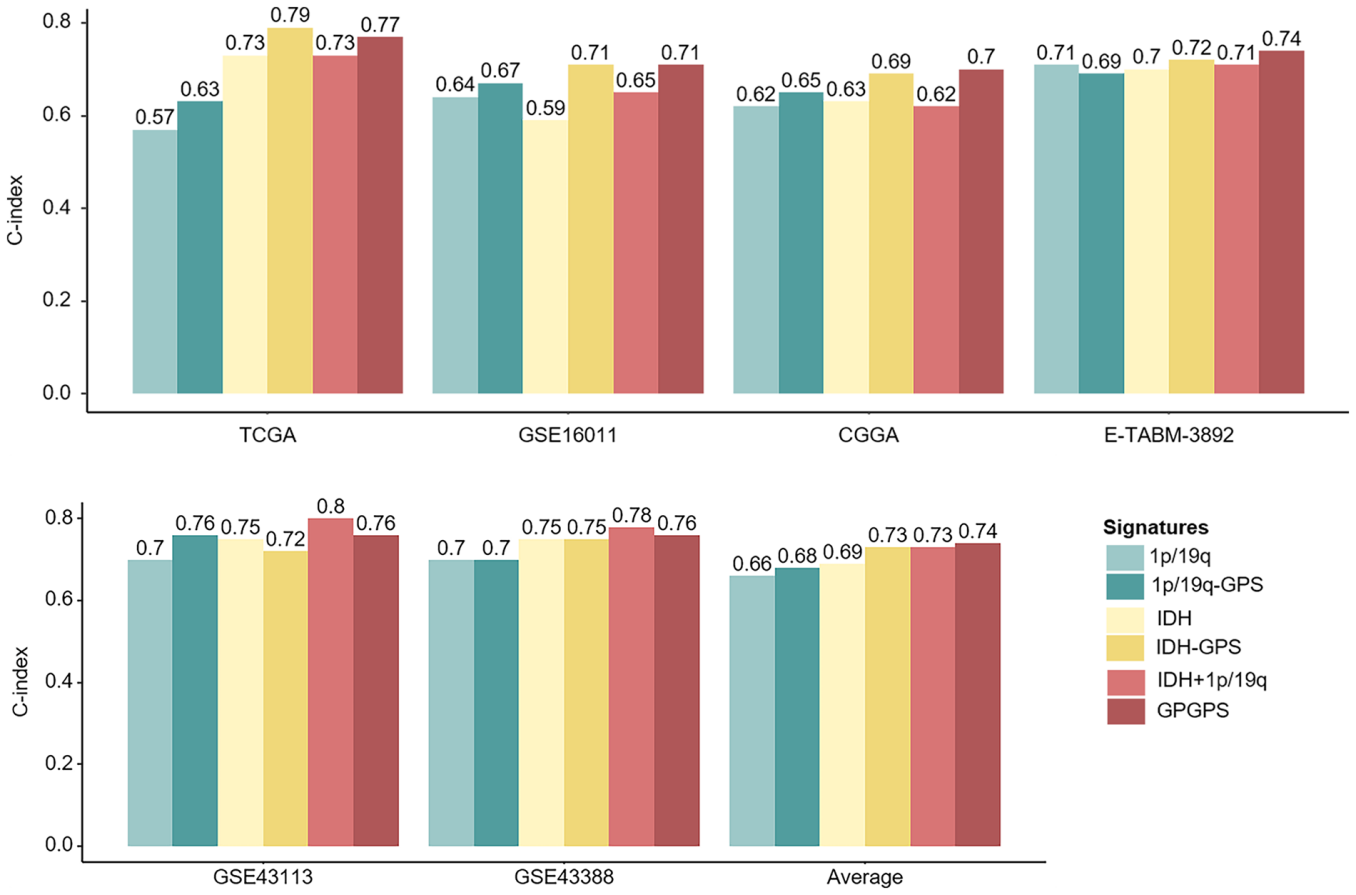
The C-index of different diagnostic markers for the six cohorts

We compared the prognostic value of GPGPS and the two basic models, *IDH*-GPS and 1p/19q-GPS, as well as the two single markers *IDH* mutation and 1p/19q-cd. Our result showed that generally the C-index of gene pairs was higher than the single prognostic marker in almost all the cohorts and the ensemble GPS was overall superior to the single GPS (Fig. 5).

**Fig. 6.**
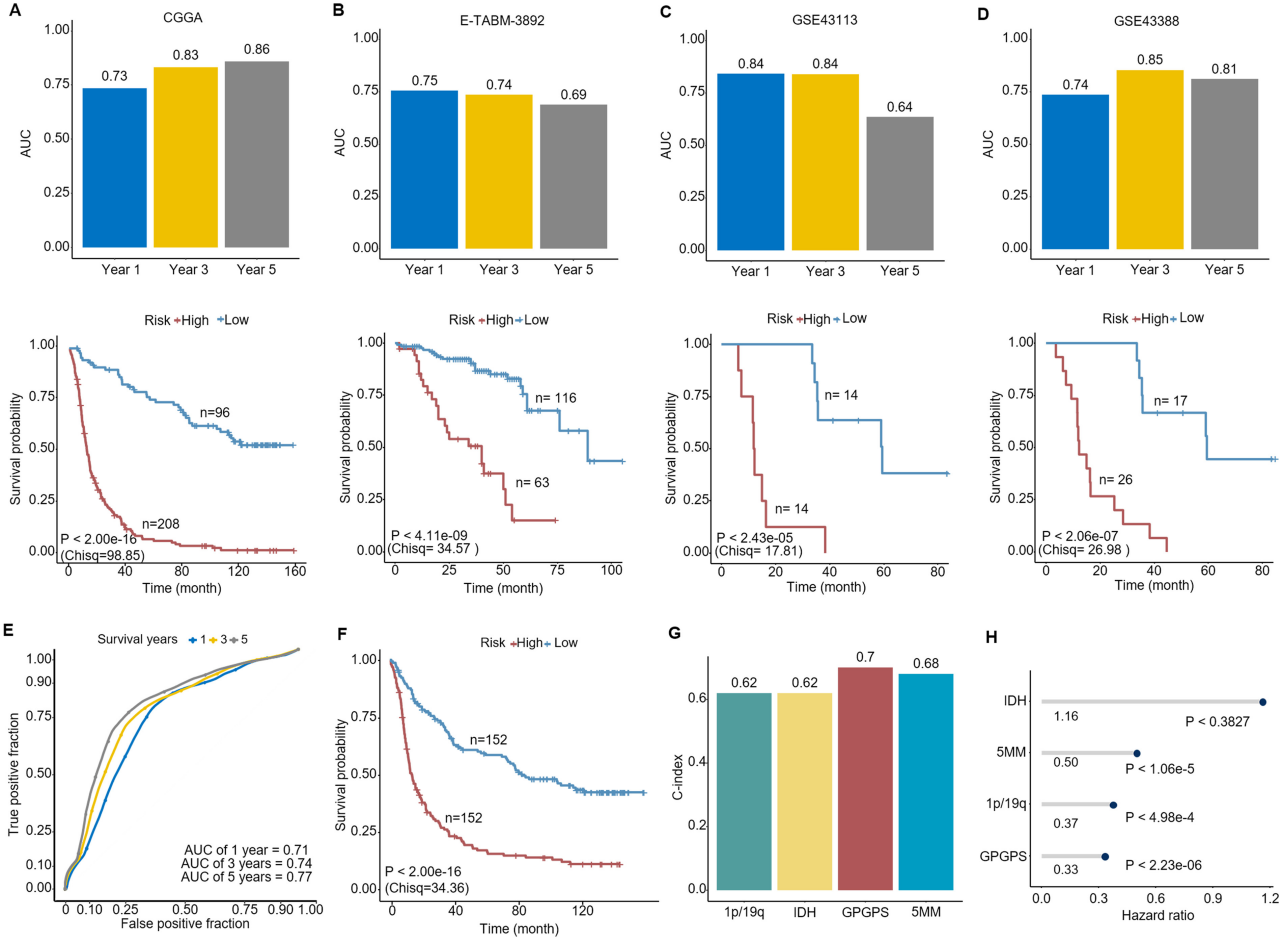
Performance evaluation and comparison of GPGPS. (**A–D**) The AUCs of 1-, 3- and 5-years OS predictions and Kaplan–Meier curves for the low-risk and high-risk patients of the ensemble model in each validation dataset. Patients with high-risk scores had poor prognosis in terms of OS. (**E**) The ROC curves of 1-, 3- and 5-years OS predictions of the m5C methyltransferases model. (**F**) Kaplan–Meier analysis of OS for the low-risk and high-risk patients of the m5C methyltransferases model. C-index (**G**) and the HRs (**H**) of the four models in the Cox proportional hazards regression analysis

In addition, the average AUCs of the four validation sets for GPGPS achieved 0.77, 0.82 and 0.75 for 1-, 3- and 5-year OS analysis, respectively (Fig. 6A–D). The distribution of the Kaplan–Meier curve showed that GPGPS was able to clearly distinguish the high- and low-risk groups (Fig. 6A–D).

Numerous studies have found that aberrant expression of RNA: m5C methyltransferases play an important role in tumor growth and development. According to a recent study on gliomas, five genes related to m5C methyltransferase were identified to construct a risk signature (5MM) and were used to predict glioma prognosis (Wang *et al.*, 2020), including *NOP2*, *NSUN4*, *NSUN5*, *NSUN6* and *NSUN7*. Methylation data of the five genes were used to quantify and discriminate good and poor prognoses. Based on the CGGA cohort, 5MM yielded AUCs of 0.71, 0.74 and 0.77 for the 1-, 3- and 5-year survival analysis (Fig. 6E and F), which is consistently less than the result of GPGPS (Fig. 6A). We also calculated and compared the C-index and HR of GPGPS and 5MM. The C-index of 5MM was 0.68, which was also second to GPGPS (Fig. 6G). The HR of gpGPS was as low as 0.33, which was less than the other three prognostic markers (1.16, 0.50 and 0.37; Fig. 6H). Consequently, we concluded that the prognostic efficacy of our ensemble model was stable and accurate.

## 4 Discussion

*IDH* mutation and 1p/19q-cd have been proven to be associated with the prognosis of glioma patients (Zhao *et al.*, 2021). In this study, we assumed that the statuses of *IDH* mutation and 1p/19q-cd coordinate the relative expression of gene pairs in glioma. Gene pair signatures were separately conducted for *IDH* mutation and 1p/19q-cd. Based on these gene pairs, we established an ensemble risk-score model, GPGPS, considering the effects of both *IDH* mutation and 1p/19q-cd to predict the diagnostic outcome of patients with glioma. GPGPS demonstrated a high performance with an average C-index of 0.74 and an average AUC of 0.82 in the 3-year OS predictions on three independent cohorts.

Our study took advantage of genome information and projected it to the transcriptome data, which improves the prognostic value. Using the reverse gene pairs may unveil potential links between genetic variation and transcriptome response, suggesting molecular and regulatory mechanisms underlying diseases, such as perturbed gene expression order between a pair of genes in specific biological processes. Notably, nearly all the genes involved in GPGPS have been reported to be associated with glioma prognosis. For instance, *RBP1* and *EMP3* participated in four and three gene pairs, respectively, and their dysregulation mechanisms in gliomas have been validated both experimentally and computationally (see Supplementary Material).

Our result showed that the reverse gene pairs are qualified to represent the genetic variations, such as mutation and chromosome co-deletion, revealing that iPAGE is an effective method for the projection from genetic variation to transcriptomic changes. Using iPAGE, the genetic variations were simultaneously projected into the same dimensional space at the expression level, transforming diverse omics features onto the same scale. Dissecting the regulatory relationships of disease-related genome alterations at the transcriptome-wide level is pivotal in understanding the physiological mechanism of diseases. Moreover, attempting to combine the strengths of different models is attractive and basically ensembling two or more models could improve the performance. Hence, other genome alterations, such as differential methylation (Zheng *et al.*, 2021) and single-nucleotide polymorphism, could also be taken into account to improve the prognostic accuracy. In fact, expression quantitative trait locus is a common method used for revealing gene regulatory relationships (Zheng *et al.*, 2020).

Consequently, we proposed an effective strategy to link disease-associated genetic variants to putative target gene pairs at the expression level, which is a projection from genome to transcriptome. GPGPS is a robust prognostic gene pair signature for glioma ensembling IDH mutation and 1p/19q-cd. Although the gene pairs were significantly associated with survival in multiple independent cohorts and several of the genes have been reported to be functional in gliomas, further experiments are needed to study the function and mechanism of these genes. Besides, GPGPS is a powerful strategy in understanding and identifying candidate causative molecules underlying various diseases, not limited to coding genes, but also non-coding RNAs, proteins, microbes and metabolites (Li *et al.*, 2020; Liu *et al.*, 2020, 2021; Song *et al.*, 2021; Yang *et al.*, 2022).

## Supplementary Material

btac850_Supplementary_Data

## Data Availability

The datasets used and/or analyzed during the current study are available from the corresponding author on reasonable request. The data underlying this article are available in the article and in its online supplementary material.
