# Supplementary material for: GPGPS: a robust prognostic gene pair signature of glioma ensembling IDH mutation and 1p/19q co-deletion
Source: Bioinformatics. 2023 Jan 13;39(1):btac850. doi: 10.1093/bioinformatics/btac850 (PMC9843586; doi:10.1093/bioinformatics/btac850)
Supplement: btac850_Supplementary_Data [file btac850_supplementary_data.docx]

**GPGPS: a robust prognostic** **gene pair signature of** **glioma ensembling** ***IDH* mutation and 1p/19q co-deletion**

Lixin Cheng^1#^*, Haonan Wu^1,2#^, Xubin Zheng^1,3^, Ning Zhang^4^, Pengfei Zhao^1,2^, Ran Wang^1,3^, Qiong Wu^5^, Tao Liu^6^, Xiaojun Yang^4^, Qingshan Geng^1,2^*

^1^ Shenzhen People's Hospital, First Affiliated Hospital of Southern University of Science and Technology, Second Clinical Medicine College of Jinan University, Shenzhen 518020, China

^2^ Department of Geriatrics, Shenzhen Clinical Research Center for Aging, Shenzhen, China

^3^ Department of Computer Science and Engineering, The Chinese University of Hong Kong, Shatin, New Territories, Hong Kong

^4^ Guangdong Provincial Key Laboratory of Infectious Disease and Molecular Immunopathology, Shantou University Medical College, Shantou 515041, China

^5^ Hong Kong Genome Institute, Hong Kong

^6^ International Digital Economy Academy, Shenzhen 518020, China

# These authors contribute equally to this work.

* To whom correspondence should be addressed. Lixin Cheng, Email: [easonlcheng@gmail.com](mailto:easonlcheng@gmail.com) or Qiangshan Geng, Email: [gengqingshan@szhospital.com](mailto:gengqingshan@szhospital.com)

**Supplementary material**

*RBP1* and *EMP3* participated in four and three gene pairs, respectively, and their dysregulation mechanisms in gliomas have been validated both experimentally and computationally. The promoter of *RBP1* tends to be hypermethylated in almost all the *IDH* mutant gliomas (either *IDH1* or *IDH2*) [1]. Survival analysis showed that the hypermethylation or low expression of *RBP1* is associated with the favorable prognosis of patients with gliomas [2]. *EMP3* is a member of the peripheral myelin protein 22-kDa (PMP22) gene family, which is reported as a tumor suppressor functioning in cell proliferation and cell-cell interactions. *EMP3* shows frequent promoter methylation in high-grade astrocytomas and neuroblastomas. Recent bioinformatics analysis also found that *EMP3* was correlated with GBM survival and could be used to estimate glioma patient prognosis [3, 4]. For the genes paired with *RBP1* and *EMP3*, a majority of them are also implemented in glioma-related pathways. *PCDH15* inhibits Wnt signaling pathway in glioblastoma progression [5]. *ATOH8* is a basic-helix-loop-helix transcription factor, whose homolog in mouse has been demonstrated to regulate neuronal versus glial fate [6]. *EYA1* drives the growth of medulloblastoma and functions as a key component of the Shh transcriptional network in oncogenesis[7]. *MYC*-mediated overexpression of *PNO1* leads to glioma progression by activating THBS1/FAK/Akt signaling [8].

Another gene pair, *IGFBP2* and *DLL3*, *DLL3* is universally expressed in the *IDH* mutant and 1p/19q co-deleted gliomas, with the majority showing highly homogeneous and intense expression, whereas the relatively lower *IGFBP2* patients frequently have mutations of *IDH1* and 1p/19q co-deletion [9, 10]. *IGFBP2* was involved in the immunosuppressive response and synergistic with several immunosuppressive members, acting as a new biomarker and potential therapeutic target for glioma immunotherapy [10].

For the gene pair *USP1* and Transmembrane protein 97 (*TMEM97*) derived from 1p/19q-GPS, *USP1* was reported to participate in a β-catenin–*USP1*-*EZH2* axis orchestrating the hyperactivated β-catenin signaling and *EZH2*-mediated epigenetic gene silencing, which represents a critical mechanism for glioma tumorigenesis [11]. *TMEM97* (also named sigma2 receptor) is an endoplasmic reticulum-resident membrane protein that is associated with meningioma. The function of the sigma2 receptor was studied through high-affinity ligands and appeared to be linked to neurodegenerative diseases and cancer development [12]. Therefore, understanding the roles and mechanisms of these gene pairs will provide novel therapeutic strategies in glioma. Our result and these pertinent literatures demonstrate the gene pairs in GPGPS are the transcriptome response of genetic effects in gliomas.

**References**

1. Chou, A.P., et al., *Identification of retinol binding protein 1 promoter hypermethylation in isocitrate dehydrogenase 1 and 2 mutant gliomas.* J Natl Cancer Inst, 2012. **104**(19): p. 1458-69.

2. Liu, Y., et al., *Methylation associated genes contribute to the favorable prognosis of gliomas with isocitrate dehydrogenase 1 mutation.* Am J Cancer Res, 2015. **5**(9): p. 2745-55.

3. Lin, S., et al., *Prognosis Analysis and Validation of m(6)A Signature and Tumor Immune Microenvironment in Glioma.* Front Oncol, 2020. **10**: p. 541401.

4. Gao, Y.F., et al., *PPIC, EMP3 and CHI3L1 Are Novel Prognostic Markers for High Grade Glioma.* Int J Mol Sci, 2016. **17**(11).

5. Han, M., et al., *Interfering with long non-coding RNA MIR22HG processing inhibits glioblastoma progression through suppression of Wnt/beta-catenin signalling.* Brain, 2020. **143**(2): p. 512-530.

6. Ducray, F., et al., *Anaplastic oligodendrogliomas with 1p19q codeletion have a proneural gene expression profile.* Mol Cancer, 2008. **7**: p. 41.

7. Eisner, A., et al., *The Eya1 phosphatase promotes Shh signaling during hindbrain development and oncogenesis.* Dev Cell, 2015. **33**(1): p. 22-35.

8. Chen, X., et al., *MYC-mediated upregulation of PNO1 promotes glioma tumorigenesis by activating THBS1/FAK/Akt signaling.* Cell Death Dis, 2021. **12**(3): p. 244.

9. Cai, J., et al., *Immune heterogeneity and clinicopathologic characterization of IGFBP2 in 2447 glioma samples.* Oncoimmunology, 2018. **7**(5): p. e1426516.

10. Spino, M., et al., *Cell Surface Notch Ligand DLL3 is a Therapeutic Target in Isocitrate Dehydrogenase-mutant Glioma.* Clin Cancer Res, 2019. **25**(4): p. 1261-1271.

11. Ma, L., et al., *Aberrant Activation of beta-Catenin Signaling Drives Glioma Tumorigenesis via USP1-Mediated Stabilization of EZH2.* Cancer Res, 2019. **79**(1): p. 72-85.

12. Liu, C.C., et al., *Sigma-2 receptor/TMEM97 agonist PB221 as an alternative drug for brain tumor.* BMC Cancer, 2019. **19**(1): p. 473.

**Supplementary figures**


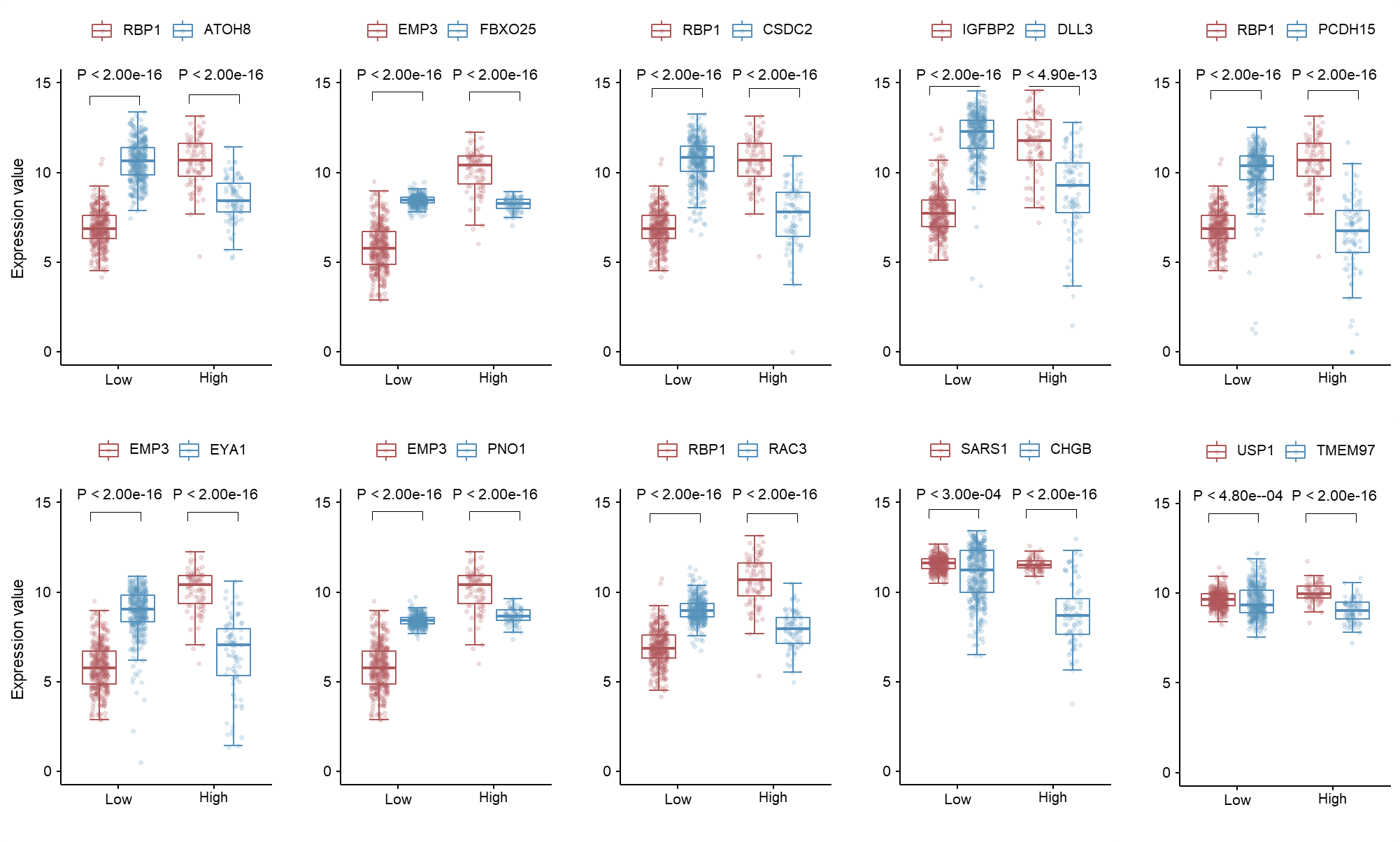


**Figure S1**. Expression pattern of the ten gene pairs included in GPGPS.
